# Double Lung Transplantation Bridged With Direct Central Ambulatory Veno-Arterial Extracorporeal Membrane Oxygenation in a Pulmonary Hypertension Patient With Extreme Mediastinal Shift

**DOI:** 10.7759/cureus.23070

**Published:** 2022-03-11

**Authors:** Samra Haroon Lodhi, Sravanthi Nandavaram, Raj Malyala, Michael Anstead, Suresh Keshavamurthy

**Affiliations:** 1 Pulmonary, Critical Care and Sleep Medicine, University of Kentucky, Lexington, USA; 2 Cardiothoracic Surgery, University of Kentucky, Lexington, USA

**Keywords:** bronchiectasis, extreme mediastinal shift, extracorporeal membrane oxygenation, pulmonary hypertension, lung transplantation

## Abstract

Lung transplantation is increasingly being performed for end-stage lung disease in patients with bronchiectasis and pulmonary hypertension. Outcomes of bilateral lung transplantation (BLT) are better in patients with pulmonary hypertension, whereas single lung transplant remains a controversy in bronchiectasis with fear of infections from the residual diseased lung. However, in patients with adhesions and extreme structural changes due to severe disease, BLT may be considered technically challenging. We describe a case of successful management of a patient with bronchiectasis-induced lung disease causing extreme mediastinal shift with a BLT. The patient was successfully bridged to transplant with central veno-arterial extracorporeal membrane oxygenation (VA-ECMO) for acute decompensated pulmonary hypertension while awaiting transplantation.

## Introduction

Lung transplantation is a therapeutic option for end-stage lung disease, including pulmonary arterial hypertension, and has increasingly been performed following the proven success of a combined heart-lung transplant [1]. Bilateral lung transplantation (BLT) has shown to have better outcomes in pulmonary hypertension (PH) patients in terms of reduction of pulmonary arterial pressures and right heart recovery [2]. A single-center study showed a 33% prevalence of PH in bronchiectasis [3]. BLT is preferred in bronchiectasis as the residual diseased lung during a single lung transplant may result in recurrent respiratory infections and unfavorable outcomes [4]. Bronchiectasis can in severe cases cause adhesions altering the lung’s architecture to the point of causing physical deviations in the structure of the mediastinum. Such changes make a BLT technically challenging and may force a single lung transplant instead of the preferred approach.

## Case presentation

A 53-year-old female with non-cystic fibrosis bronchiectasis, chronic respiratory failure on four liters of supplemental home oxygen, and severe secondary PH with right ventricular (RV) dysfunction was listed for BLT at our center. The patient suffered from an extreme mediastinal shift with a right-sided deviation of the heart because of disease-related right lung parenchymal destruction. Transthoracic echocardiogram (TTE) during transplant evaluation showed normal left ventricular (LV) function with ejection fraction (EF) of 50%-55%. RV was severely dilated with moderately reduced systolic function, and tricuspid annular plane systolic excursion (TAPSE) was observed. Right heart catheterization showed an elevated pulmonary artery systolic pressure (PASP) of 84 mmHg (normal 18-25 mmHg), mean pulmonary artery pressure (mPAP) of 49 mmHg (normal 12-16 mmHg), and pulmonary vascular resistance (PVR) of 8.4 Wood Units (WU) (>3 WU considered pulmonary arterial hypertension). The pulmonary capillary wedge pressure (PCWP) was normal at 8 mmHg. 

**Figure 1:**
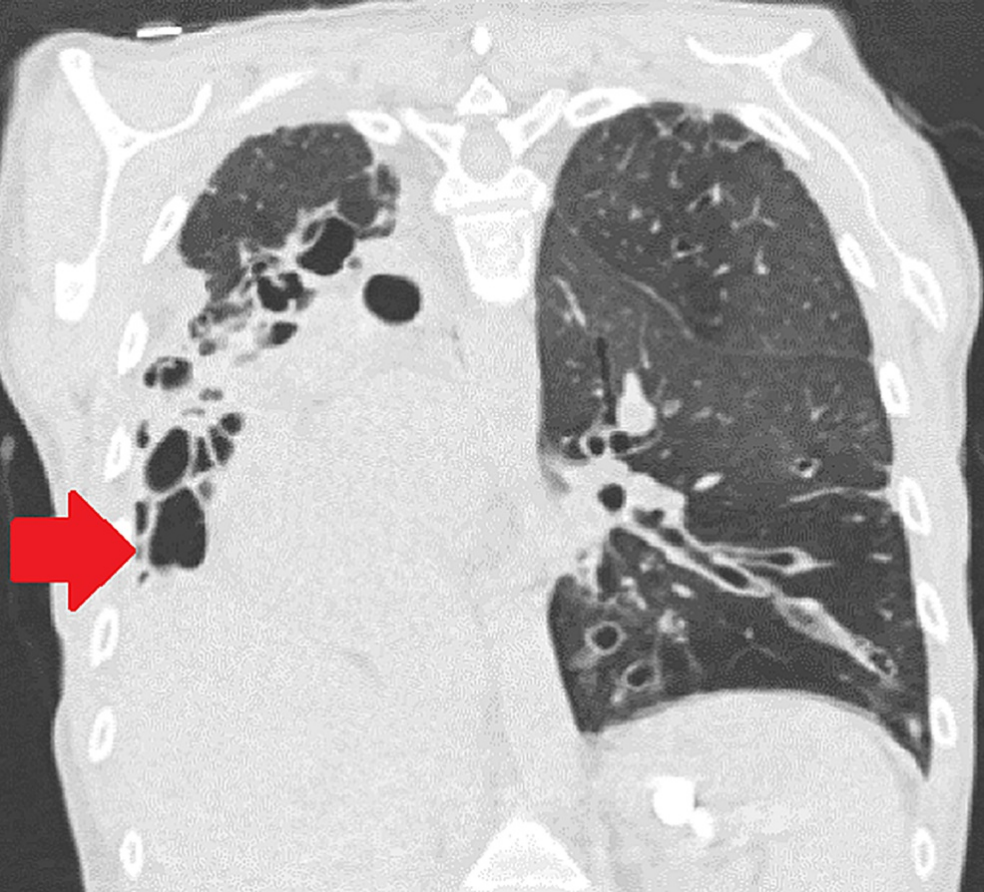
CT chest coronal view shows lower lobe predominant bronchiectasis, heart deviated to the right with extreme right lung destruction (red arrow)

**Figure 2:**
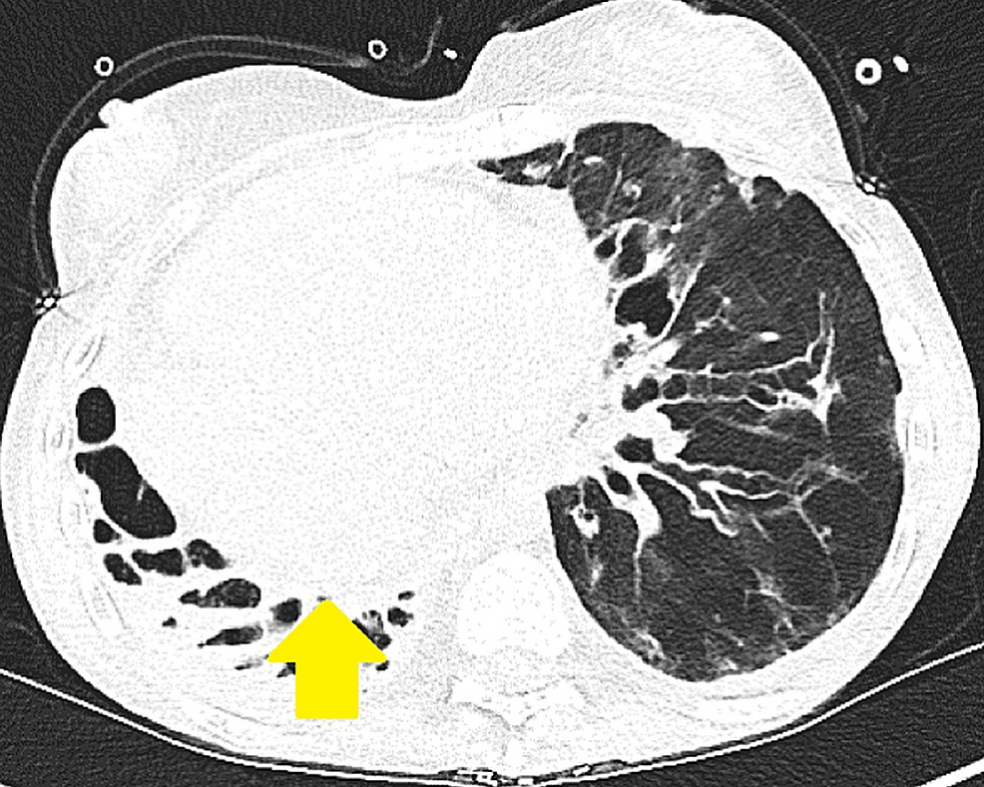
CT chest axial view shows bronchiectasis with extreme right-sided deviated mediastinal structures (yellow arrow)

While on the waitlist, the patient was admitted to the hospital for worsening shortness of breath, lower extremity swelling, and hypoxia requiring high flow oxygen supplementation. On presentation, her vitals included a temperature of 97.5 F, blood pressure of 102/69 mmHg, heart rate of 78 beats per minute, and respiratory rate of 17 breaths per minute. The patient was placed on a high flow with 20 L oxygen via nasal cannula with 50% FiO_2_. A lower extremity deep venous thrombus was ruled out on Doppler ultrasound. Brain natriuretic peptide (BNP) was elevated at 2,810 pg/mL. A TTE showed severely dilated RV with severely reduced systolic function, estimated RVSP of more than 90 mmHg, and moderate tricuspid regurgitation. LV cavity was small with normal systolic function and LVEF of more than 60%. The interventricular septum was flattened. Chest computed tomography (CT) showed stable lower lobe predominant bronchiectasis (Figures 1, 2).

With no active disease on the chest imaging and insignificant laboratory findings, infection was ruled out. Despite being administered diuretics for right-sided heart failure, the patient decompensated necessitating invasive mechanical ventilation, and her hemodynamics deteriorated requiring vasopressor support. Veno-arterial extracorporeal membrane oxygenation (VA ECMO) was initiated at the bedside for a refractory shock as a bridge to transplant. A 24 Fr outflow cannula was placed in the right femoral vein, a 17 Fr inflow cannula was placed in the right femoral artery with a 6 Fr distal perfusion cannula in the right superficial femoral artery.

**Figure 3:**
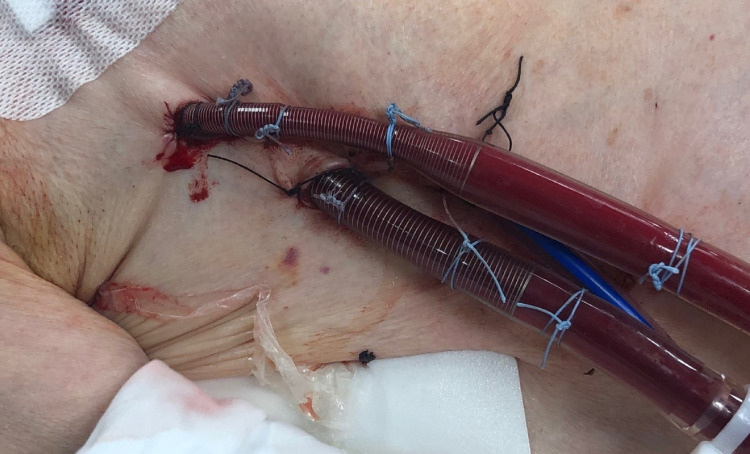
Direct central ambulatory VA ECMO shows utilizing a minimally invasive approach via right anterior thoracotomy in the right second intercostal space

The patient’s hemodynamics improved after the initiation of VA ECMO. The following day she was transitioned to direct central ambulatory VA ECMO, utilizing a minimally invasive approach via right anterior thoracotomy in the right second intercostal space (Figure 3). Her distorted anatomy with pleural adhesions from bronchiectasis helped expose both aorta and right atrium (RA) on opening the pericardium. A 32 Fr malleable outflow cannula was placed in the RA and a 20 Fr inflow cannula was placed in the ascending aorta; both cannulas were tunneled through the intercostal space. The patient was maintained on central VA ECMO with a flow of 4.8 LPM, a sweep of 5 LPM, and FiO_2_ of 100%. Six days later upon the availability of suitable donor lungs, she underwent bilateral sequential lung transplantation.

The donor was a 54-year-old nonsmoker with a history of cerebrovascular accident, on pressure regulated volume control (PRVC) ventilation. Lung compliance was 45 with PaO_2_ 476 mmHg on oxygen challenge. CT scan of the chest showed bilateral lower lobe atelectasis. Bronchoscopy revealed normal right and left bronchial anatomy and mucosa without any secretions. Bronchoalveolar lavage was positive for *Streptococcus salivarius, methicillin-sensitive Staphylococcus aureus, and Streptococcus parasanguinius*. The donor was maintained on piperacillin-tazobactam.

Perioperative transesophageal echocardiogram (TEE) of the recipient showed decreased LV function with 40% LVEF, severely dilated RV with severely reduced RV systolic function with 10% EF. Both right and left atria were dilated, and no PFO was noted. Bilateral sequential lung transplantation was performed on VA ECMO via clamshell thoracosternotomy. The left lung was implanted first followed by the right lung. The patient was successfully weaned off VA ECMO, which was decannulated and the chest closed in standard fashion. There was a concern during chest closure with higher peak airway pressures which was managed by transitioning to the ICU ventilator and continuing inhaled epoprostenol. Post bilateral lung implantation, LV, and RV function markedly improved. LVEF was 45% and RVEF was 35%. Pulmonary venous flows and velocities were within normal limits. She was transferred to the ICU on epinephrine 0.04 mcg/kg/min, norepinephrine 0.1 mcg/kg/min, vasopressin 0.04 units per minute and milrinone 0.25 mcg/kg/min. She was maintained on pressure control mode ventilation with a pressure of 20 cm of H_2_O, PEEP of 8 cm of H_2_O, and FiO_2_ 60%. Lung compliance was between 30 and 35 mm/cm H_2_O.

**Figure 4:**
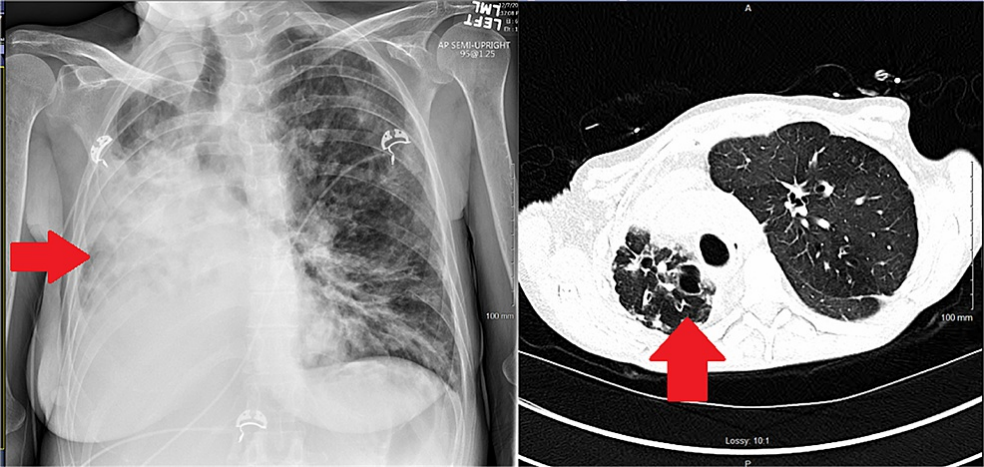
Preoperative imaging with an extreme mediastinal shift (red arrows)

**Figure 5:**
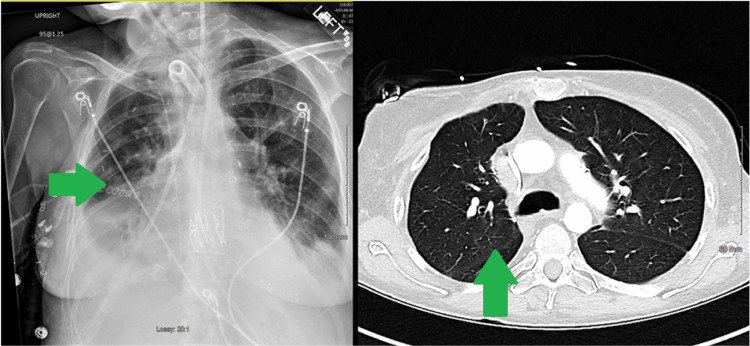
Post-transplant imaging with the resolution of mediastinal shift (green arrows)

Over the ensuing 72 hours, the oxygenation improved, vasopressor requirement decreased, and her hepatic parameters improved; she was deemed too weak and fragile to tolerate extubation and consequently underwent an elective percutaneous tracheostomy following which she was gradually liberated from mechanical ventilation. Her postoperative imaging showed a return to normal cardiac position compared to the extreme rightward shift preoperatively (Figures 4, 5). A follow-up echocardiogram a few months after transplant revealed normal RV size and function. LV systolic function was normal, with an estimated EF > 55%.

## Discussion

Lung transplantation is a therapeutic option for end-stage lung disease including PH and is being increasingly performed following the initial success of heart-lung transplantation for end-stage PH. Single- (SLT) and BLTs have mostly replaced heart-lung transplants because the PVR decreases rapidly allowing the RV to recover [2,5,6]. Studies have shown marked improvement of mPAP, PASP, and right heart dysfunction after lung transplant [7]. Studies comparing SLT and BLT have shown BLT to have a survival advantage [2]. One study reported SLT recipients with underlying PH to have less functional recovery including less reduction in mPAP [8].

SLT is particularly controversial in patients with bronchiectasis as leaving the diseased lung allows infectious sequelae [4]. Our patient was suffering from an extreme mediastinal shift due to bronchiectasis-related destruction of the right lung parenchyma. While being evaluated for lung transplantation the patient was being considered for a left single transplant as a double lung transplant would require technically complex lung removal and implantation with increased bleeding risk. Earlier case reports have described the management of similar cases with SLT, and contralateral pneumonectomy given technical difficulty [9,10]. Recent literature describes cases of BLT in asymmetric thoraces with graft reduction to fit the volume-reduced hemithorax [4,11]. Yue et al. described six cases of BLT in patients with asymmetric thoraces at their center [11]. Four patients required graft reduction while two patients were managed with intraoperative resection of mediastinal adhesions and no graft reduction, as with our patient. This approach allowed for maximal preservation of donor lung function.

In patients with acutely decompensated PH leading to shock, extracorporeal life support (ECLS) technology can temporarily stabilize hemodynamics, acting as a bridge to lung transplantation [12]. Central ECMO allows early ambulation and participation in physical therapy as candidates await transplantation [13]. Although less invasive, peripheral ECMO may induce vascular damage and limb ischemia over prolonged periods [13].

In transplanted patients, postoperative dynamic RV and LV conformational changes can result in hemodynamic instability and primary graft dysfunction (PGD) which is strongly associated with recipient PH (primary or secondary) [14]. Therefore, certain considerations are made pre-and post-transplant to prevent PGD. These include factors related to donor lung quality and intensive care support for recipients.

Donor lung quality plays a crucial role in post-transplant recovery, especially in the first 24 hours; thereafter, underlying recipient factors like PH become more important [15]. Standard objective donor lung assessment includes age, oxygenation, imaging, ABO compatibility, size, and absence of major chest trauma or infection [15,16]. Lung compliance is often overlooked as a variable for the quality assessment of a donor’s lungs. Studies have shown a significant correlation of lung compliance with reduced duration of postoperative mechanical ventilation [17].

At our center, we utilize dynamic compliance of 30 or greater for lung acceptance. Though such cut-off can further limit the donor pool, we have also noted an association with a shorter duration of postoperative ventilatory times with better donor dynamic compliance.

Some patients, especially with long-standing PH, RV hypertrophy, and dysfunction, might need prolonged vasopressor support and a certain proportion of patients might need ECLS post-transplant to allow the institution of more lung-protective strategies [18,19]. VA ECMO ensures controlled perfusion to the graft and provides time for the LV to adapt to the new PVR and cavity changes. ECMO can serve both as a bridge to transplant and as a bridge to recovery [20].

## Conclusions

Double lung transplant is associated with better outcomes in end-stage lung disease secondary to bronchiectasis and PH. Although surgically challenging, a double lung transplant can be performed safely in a patient with an extreme mediastinal shift. We hypothesize that compliant lungs might aid early right heart recovery from the effects of PH and reduce the post-op ventilator time and the need for ECLS. Acknowledging that a definite cut-off will further limit the donor pool, we suggest considering lung compliance in conjunction with other variables. However, larger-scale studies are needed to validate this hypothesis. ECMO is a valuable tool when introduced timely to provide cardiorespiratory support while awaiting surgery and also after surgery to allow the heart to adapt to the new hemodynamic parameters.
